# Supplementary material for: Functional analysis of cyclic diguanylate-modulating proteins in Vibrio fischeri
Source: mSystems. 2024 Oct 22;9(11):e00956-24. doi: 10.1128/msystems.00956-24 (PMC11575326; doi:10.1128/msystems.00956-24)
Supplement: Supplemental Figures — Figures S1-S3. [file msystems.00956-24-s0001.pdf]

A

Group	Protein	Motility: TBS	Motility: Mg	Motility: Ca
DGCs (GGDEF)	0596 (CdgG)			
	0989 (MifA)	-	-	-
	1200	-	-	-
	1245			
	1350	-		
	1515			
	1561	-	-	-
	1639 (CasA)	-	-	-
	2261	-	-	
	2362	-	-	-
	A0056			
	A0057	-	-	-
	A0152	-	-	-
	A0155	-	-	-
	A0276	-	-	-
	A0323	-	-	-
	A0342	-	-	-
	A0343	-	-	-
	A0368	-	-	-
	A0381	-	-	-
	A0398	-	-	-
	A0476			
	A0567	-	-	-
	A0692	-	-	-
	A0796			
	A0959 (MifB)	-	-	-
	A0976			+
	A1012	-	-	-
	QrgB	-	-	-
PDEs (EAL)	0087			
	0091			
	1603	+		+
	2480	+		+
	A0344			+
	A0526			
	A0551	+		
	A0706			
	A0879			
	A1014 (PdeV)			
	A1038 (BinA)	+		+
	A1076			
	VC1086	+		+
PDEs (HD-GYP)	1367			
	A0506	+		+
DGC/PDEs	0094	+		+
	0494	+		+
	0985	-		-
	A0244			
	A0475			
Degenerate	0355			
	A0216			
	A1166 (LapD)	-	-	

B

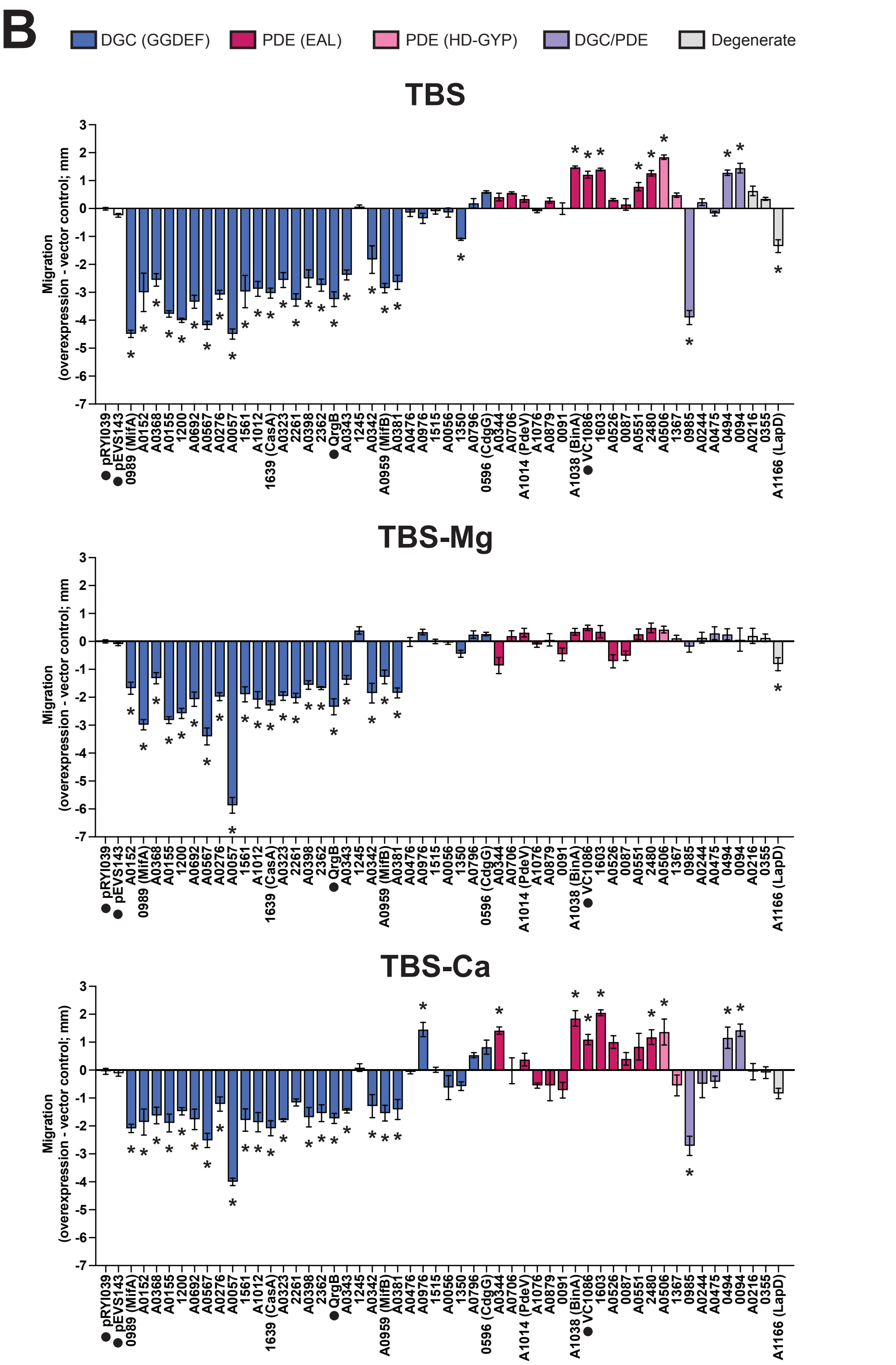

FIG S1. Many predicted V. fischeri DGCs and PDEs impact swimming motility when overexpressed. A. Summary of motility results for overexpression of indicated proteins in TBS, TBS-Mg, and TBS-Ca soft (0.3%) agar. Olive coloring indicates phenotypes expected from elevated c-di-GMP, whereas orange indicates phenotypes expected from reduced c-di-GMP. White indicates no significant change. **B.** Quantification of migration through TBS, TBS-Mg, and TBS-Ca soft (0.3%) agar for *V. fischeri* strains overexpressing the indicated proteins relative to the pRYI039 empty vector control. TBS data is the same as represented in FIG 2B. For each strain, n = 4-12 biological replicates (33-36 for controls). One-way analysis of variance (ANOVA) was used for statistical analysis, each bar represents the means of biological replicates, error bars represent standard errors of the mean, asterisks represent significance relative to the pRYI039 empty vector control (*, $P < 0.05$), and numbers represent VF_ locus tags (e.g., VF_0087, VF_A0056, etc.); negative controls pRYI039 and pEVS143 as well as non-*V. fischeri* controls QrgB and VC1086 are also listed and indicated with a black dot.

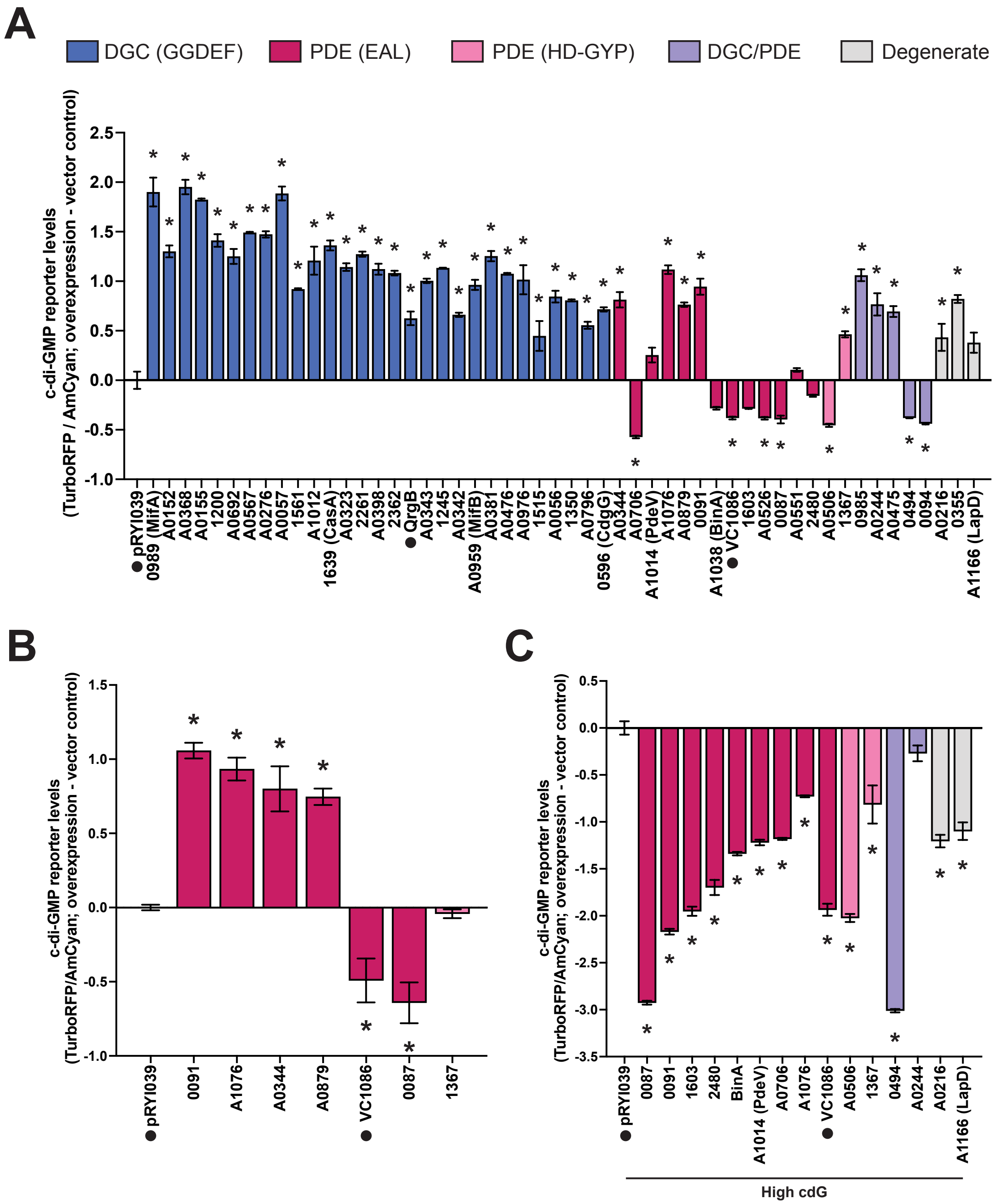

D

Protein	cdG	CR	Mot
0596	+		
0989 (MifA)	+	+	-
1200	+	+	-
1245	+		
1350	+		-
1515	+		
1561	+	+	-
1639 (CasA)	+	+	-
2261	+	+	-
2362	+	+	-
A0056	+		
A0057	+	+	-
A0152	+	+	-
A0155	+	+	-
A0276	+	+	-
A0323	+	+	-
A0342	+		-
A0343	+	+	-
A0368	+	+	-
A0381	+		-
A0398	+	+	-
A0476	+		
A0567	+	+	-
A0692	+	+	-
A0796	+		
A0959 (MifB)	+		-
A0976	+		
A1012	+	+	-
QrgB	+	+	-
0087	-	-	
0091	+	-	
1603		-	+
2480		-	+
A0344	+		
A0526	-	-	
A0551		-	+
A0706	-		
A0879	+		
A1014 (PdeV)			
A1038 (BinA)		-	+
A1076	+		
VC1086	-	-	+
1367	+	-	
A0506	-	-	+
0094	-	-	+
0494		-	+
0985	+	+	-
A0244	+		
A0475	+		
0355	+		
A0216	+		
A1166 (LapD)			-

FIG S2. C-di-GMP biosensor activity. Quantification of c-di-GMP concentration for *V. fischeri* strains overexpressing the indicated proteins using the pFY4535 c-di-GMP reporter plasmid. **A.** Assays performed in 96-well format. Values are relative to the pRYI039 empty vector control. For each strain, n = 3-6 (15 for control) biological replicates. **B.** Assays performed in non-96-well format. Values are relative to the pRYI039 empty vector control. For each strain, n = 3-7 biological replicates (11 for control). **C.** Assays of indicated proteins overexpressed in a high c-di-GMP background. For each strain, n = 3-9 biological replicates. **D.** Integration of phenotypic data for the *V. fischeri* DGCs and PDEs. Olive coloring indicates phenotypes expected from elevated c-di-GMP, whereas red-orange indicates phenotypes expected from reduced c-di-GMP. White indicates no significant change. For A to C, constitutive AmCyan was used to normalize TurboRFP to cell density, one-way analysis of variance (ANOVA) was used for statistical analysis, each bar represents the means of biological replicates, error bars represent standard errors of the mean, asterisks represent significance relative to the pRYI039 empty vector control (*, P < 0.02). For A to D, numbers represent VF_ locus tags (e.g., VF_0087, VF_A0056, etc.); negative controls pRYI039 and pEVS143 as well as non-*V. fischeri* controls QrgB and VC1086 are also listed and indicated with a black dot.

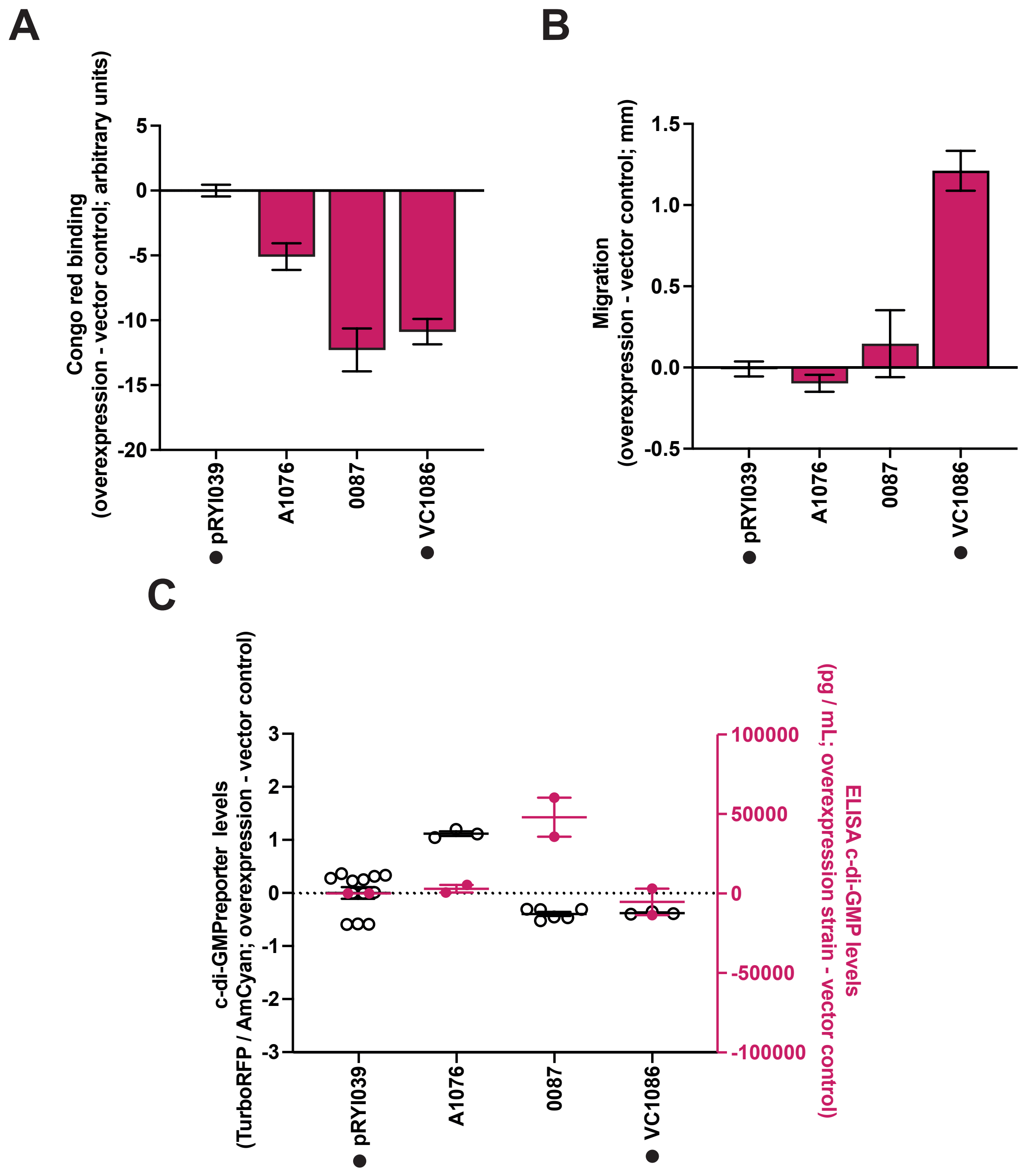

FIG. S3. C-di-GMP quantification methods do not match PDE functional characterization.
A. Quantification of Congo red binding for *V. fischeri* PDEs overexpressing the indicated proteins relative to the pRYI039 empty vector control. For each strain, n = 5 biological replicates. Each bar represents the means of biological replicates. Data are the same as those represented in FIG 2A. **B.** Quantification of migration through soft (0.3%) agar for *V. fischeri* PDEs overexpressing the indicated proteins relative to the pRYI039 empty vector control. For each strain, n = 4 biological replicates. Each bar represents the means of biological replicates. Data are the same as those represented in FIG 2B. **C.** Quantification of c-di-GMP concentration for *V. fischeri* strains overexpressing the indicated PDEs using the pFY4535 c-di-GMP reporter plasmid (left y-axis; open dots) and ELISA (right y-axis; solid dots). Values are relative to the pRYI039 empty vector control. For each strain, n = 3 (9 for controls) biological replicates, dots represent the means of technical replicates, average bars represent the means of biological replicates. C-di-GMP reporter data are the same as those represented in FIG S2A. For A-C, error bars represent standard errors of the mean and numbers represent VF_ locus tags (e.g., VF_0087, VF_A0056, etc.); negative control pRYI039 and non-*V. fischeri* control VC1086 are also listed and indicated with a black dot.
